# Self-Medication for Sleep Disorders in Tunisia: Understanding Motivations and Potential Risks

**DOI:** 10.1192/j.eurpsy.2026.12096

**Published:** 2026-07-31

**Authors:** K. Mahfoudh, S. Hamzaoui, S. Walha, M. Mrad, R. Hosni, A. Ouertani, A. Aissa, R. Jomli

**Affiliations:** Avicenne, Razi Hospital, Manouba, Tunisia

## Abstract

**Introduction:**

Sleep disorders, particularly insomnia, are a widespread concern, with many individuals turning to self-medication for relief. While self-medication may provide immediate comfort, it carries significant risks due to the absence of medical supervision. Understanding the motivations behind this behavior, the perceived risks, and the sources of information individuals rely on is essential to fostering safer health practices and improving sleep disorder management.

**Objectives:**

This study aims to explore the motivations behind self-medication for sleep disorders in Tunisia, identify the perceived risks, and investigate the information sources influencing this practice.

**Methods:**

An online cross-sectional study was conducted between August and November 2024 using a self-administered questionnaire. The survey was distributed through social media platforms, targeting adults in Tunisia. The questionnaire collected data on sociodemographic characteristics, self-reported insomnia symptoms, self-medication practices, motivations, side effects, sources of information, and knowledge of associated risks.

**Results:**

Among 287 participants, 60.98% were women and 39.02% men, with most aged 26–35 years (52.96%). Most lived in urban areas (93.38%), had a university degree (88.15%), and were employed (65.16%).

Self-medication for insomnia was reported by 23.69%, mainly due to ease of access (55.88%), preference for natural remedies (52.94%), and prior positive experiences (39.7%). Adverse effects occurred in 28.45%, mostly mood changes (88%), daytime sleepiness (73%), and headaches (48%).

Regarding risks, 40.77% were unaware of dangers, while informed participants cited drug dependence (43.52%), side effects (40.58%), and long-term health issues (27.05%). Sources of information included the internet (80.88%), physicians (69.11%), family/friends (42.64%), and pharmacists (35.29%). Notably, 61% never received professional advice, while 51.6% used non-pharmacological strategies like relaxation or meditation.

**Conclusions:**

Self-medication for sleep disorders is common in Tunisia, driven by accessibility and natural remedy preferences. While some are aware of the risks, many lack professional advice. These findings emphasize the need for improved education and healthcare guidance on safe sleep management practices.

**Disclosure of Interest:**

None Declared

